# AGE AS A PREDICTOR OF BURN-RELATED OUTCOMES IN OLDER ADULTS IN LOW- AND MIDDLE-INCOME COUNTRIES

**DOI:** 10.1093/geroni/igae098.0720

**Published:** 2024-12-31

**Authors:** Claire Wang, Jieun Lee, Elizabeth Khvatova

**Affiliations:** Johns Hopkins University, Baltimore, Maryland, United States; Johns Hopkins University, Baltimore, Maryland, United States; Johns Hopkins University, Baltimore, Maryland, United States

## Abstract

While older age has been linked to worsened burn outcomes, the specific effects of age on high-risk older adult burn patients in low- and middle-income countries (LMICs) are not well understood. Utilizing the WHO Global Burn Registry data from 2017-2021, older adults (n=2,257) were stratified into three age groups at time of burn injury (>40 and ≤60; >60 and ≤80; >80). Multivariable logistic regression models were employed to evaluate the association between age groups and detailed burn outcomes (left against medical advice, discharged home without physical impairment, discharged home with physical impairment, or mortality), adjusting for potential confounders such as sex, country, percentage of total burn surface area, access to rehabilitation services, and surgical interventions during hospitalization. After adjustment, individuals ages 80+ showed a statistically significant increase in burn-related mortality/impairment compared to those in the >40 and ≤60 age group, with an adjusted odds ratio (OR) of 2.06 (95% confidence interval [CI]: 1.33-3.20). A separate analysis on mortality revealed that those 80+ had significantly higher odds of burn-related mortality compared to the youngest age group, with an OR of 3.47 (95% CI: 2.10-5.74). No significant differences were identified in impairment outcomes across the age groups. Our study reveals a marked increase in mortality risk from burns in individuals 80+ in LMICs, not observed in younger counterparts. Despite no significant age-impairment correlation, the findings indicate a need for age-tailored interventions to improve outcomes for this high-risk group, urging further research into targeted, age-specific care.

